# An unusual evolution of thyroid function after therapeutic plasma exchange in Graves’ disease with cholestatic jaundice: A case report

**DOI:** 10.1097/MD.0000000000037074

**Published:** 2024-02-23

**Authors:** Yamei Jin, Xiaohong Feng, Haixiang Ni

**Affiliations:** a Department of Endocrinology, The First Affiliated Hospital of Zhejiang Chinese Medical University (Zhejiang Provincial Hospital of Chinese Medicine), Hangzhou, Zhejiang, China.

**Keywords:** cholestatic jaundice, Graves’ disease, hyperthyroidism, methimazole, therapeutic plasma exchange

## Abstract

**Rationale:**

Methimazole (MMI) is the first-line agent in the treatment of hyperthyroidism. However, rare but severe cholestatic jaundice may occur. Therapeutic plasma exchange (TPE) may provide an alternative treatment for such patients and they received thyroidectomy/radioactive iodine ablation or continued oral anti hyperthyroidism medication immediately after TPE session in the reported literatures. The case reported here is, to our knowledge, the first to describe the long interval between anti hyperthyroidism therapy and TPE in such patients.

**Patient concerns:**

A 49-year-old Chinese woman had developed worsening jaundice 3 weeks after receiving methimazole (20 mg/day) for the treatment of hyperthyroidism secondary to Graves’ disease (GD). Additionally, she had a 2-year history of type 2 diabetes.

**Diagnosis:**

Hyperthyroidism secondary to GD, MMI-induced severe cholestatic jaundice and type 2 diabetes.

**Interventions:**

Methimazole was discontinued and the patient received 3 times of TPE, about 3-month glucocorticoid treatment, insulin administration accordingly and other conventional liver-protecting therapy.

**Outcomes:**

Her thyroid function was stabilized with small dose of thyroxine substitution and euthyroid status persisted after thyroxine discontinuation until hyperthyroidism recurred 7 months later while her cholestatic jaundice was eventually recovered by about 3-month glucocorticoid therapy.

**Lessons:**

Due to the complex interplay between liver function and thyroid hormones, there may be unusual changes of thyroid function in GD patients with severe liver injury after TPE. By this case, we want to highlight the importance of a closely following up of thyroid function in order to deliver appropriate health suggestions for patients.

## 1. Introduction

Methimazole (MMI) is the first-line agent in the treatment of hyperthyroidism and is generally well tolerated. However, rare but severe hepatotoxicity, such as cholestatic jaundice may occur.^[1,2]^ No known effective medical intervention has been existed. Therapeutic plasma exchange (TPE) is a blood purification method that can immediately remove harmful plasma constituents and has been reported to successfully treat severe cholestatic jaundice induced by MMI.^[3]^ TPE can also remove a large number of serum protein-bound thyroid hormones and has been used as one of the effective alternative therapies in severe hyperthyroidism since the 1970s.^[4]^ Thus, TPE may provide an alternative treatment for hyperthyroidism with severe drug-induced liver injury. However, there has not been any constructive recommendation on the interval between deterministic therapy of hyperthyroidism and TPE, although patients received radioactive iodine ablation or surgery immediately after TPE session in the reported literatures.^[3,5]^ Here, we present a case of a 49-year-old woman with hyperthyroidism secondary to Graves’ disease (GD), MMI-induced severe cholestatic jaundice and type 2 diabetes. She received 3 times of TPE, about 3-month glucocorticoid treatment, insulin administration accordingly and other conventional liver-protecting therapy. Curiously, her thyroid function stabilized after TPE over a long period of time in the absence of any anti hyperthyroidism treatment.

## 2. Case report

A 49-year-old Chinese woman was admitted to our hospital due to worsening jaundice for 2 months. She had a three-month history of GD which was diagnosed by elevated plasma levels of free triiodothyronine (FT3), free thyroxine (FT4) and antibodies to the thyrotropin receptor (TRAb), decreased level of thyroid stimulating hormone (TSH) in the local hospital. The initial laboratory studies showed her liver function was normal. Treatment of MMI (20 mg/day) was prescribed for hyperthyroidism but was discontinued after 3 weeks due to the symptom of generalized icterus.

**Figure 1.**
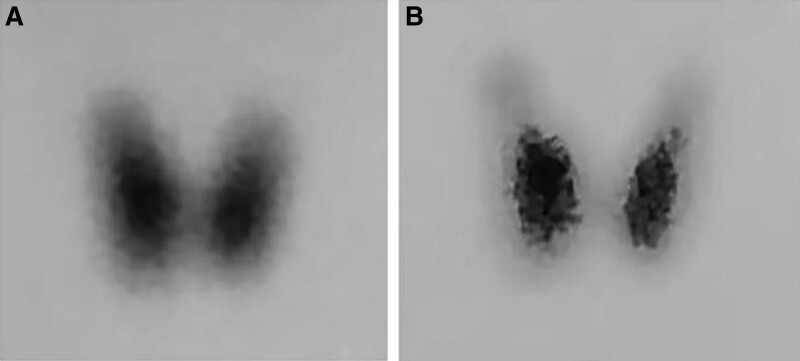
(A) Thyroid radioactive ^99^mTc uptake and scan revealing evaluated uptake before therapeutic plasma exchange of the patient. (B) Follow-up radioactive scintigraphy showing evaluated uptake of ^99^mTc when hyperthyroidism recurred.

She was firstly admitted to another hospital. Initial evaluation revealed evidence of hyperthyroidism with acute liver failure. Thyroid function test was as follows: FT3 12.15 pmol/L (normal, 2.43–6.01 pmol/L), FT4 32.92 pmol/L (normal, 9.01–19.05 pmol/L), TSH: <0.004 μIU/mL (normal, 0.350–4.940 μU/mL), TRAb 5.77 (normal, 0.00–1.75 IU/L). Radioactive ^99^mTc uptake and scan revealed 2 hours ^99^mTc uptakes was 6.31% (normal, 0.24–3.34%) (Fig. 1), consistent with Graves’ disease. Liver function test showed highly elevated levels of total bilirubin (TBil, 322.4 μmol/L), direct bilirubin (DBil, 289.9 μmol/L) and alkaline phosphatase (ALP, 235 U/L). Ursodeoxycholic acid (UDCA) was taken orally and ademetionine was transfused for liver protection. TPE was performed on the 3rd, 6th and 18th days after admission. TPE consisted of removing 1.5 volumes of plasma and replaced it with a colloid solution containing 5% albumin (Alb) during each session. FT3 decreased to 2.16 pmol/L and FT4 decreased to 8.35 pmol/L with TSH < 0.004 μIU/mL after the second plasma exchange. Radioactive iodine (^131^I) treatment plan was dropped and the patient was later started on levothyroxine (25 μg/day) and her thyroid function remained stable. However, her serum TBil and DBil were still at high levels.

Consequently, she was transferred to our hospital. In the detailed inquiry, she did have a 2-year history of type 2 diabetes and had no antidiabetic medicine therapy. She reported no alcohol or tobacco use, no blood transfusion and no histories of heart disease or viral hepatitis. Physical examination on admission revealed a heart rate of 63 beats per minute, blood pressure of 109/74 mm Hg, respiratory rate of 18 breaths per minute and a temperature of 36.7 °C, with severe icterus of the sclerae and skin. Her thyroid was diffusely enlarged while both the liver and spleen were not palpable.

Repeated laboratory workup showed FT3 and FT4 were lower than normal, at 2.08 pmol/L and 8.86 pmol/L, respectively, with TSH was 0.01 μIU/mL (Table 1). Liver function evaluations were consistent with hepatotoxicity with TBil 247.1 μmol/L, DBil 216.9 μmol/L, cholinesterase (4385 U/L, normal,5000–12,000 U/L) and Alb (32.3 g/L, normal,40–55 g/L) (Table 2). Complete blood cell count and basic chemistry and coagulation test results were unremarkable or within normal limits. Tests for hepatitis A, B, C, E viruses and for toxoplasmosis, cytomegalovirus, and Epstein-Barr viruses were all negative. Additional evaluation including autoimmune hepatitis antibodies, levels of ceruloplasmin and copper were within normal limits. Ultrasonography showed diffuse thyroid lesions and unremarkable changes of the liver, pancreas, and spleen, with no evidence of biliary ductal dilation or obstruction.

**Table 1:** Thyroid function tests during hospitalization.

Data unit	TT3 nmol/L	TT4 nmol/L	FT3 pmol/L	FT4 pmol/L	TSH μIU/mL
10/09/21	0.70	84.77	2.08	8.86	0.01
10/25/21	0.44	60.00	1.81	8.53	0.10
11/15/21	0.75	65.96	1.95	8.71	0.36

FT3 = free triiodothyronine, normal range 2.43–6.01 pmol/L, FT4 = free thyroxine, normal range 9.01–19.05 pmol/L, TSH = thyroid stimulating hormone, normal range 0.35-4.94 μIU/mL, TT3 = triiodothyronine, normal range 0.98-2.33 nmol/L, TT4 = tetraiodothyronine, normal range 62.68–150.84 nmol/L.

**Table 2:** Liver function tests during hospitalization.

Data unit	TBil μmol/L	DBil μmol/L	AST IU/L	ALT IU/L	ALP IU/L	γ-GT IU/L	CHE IU/L	TP g/L	Alb g/L
10/09/21	247.1	216.9	28	36	121	63	4385	51.9	32.3
10/12/21	266.3	225.9	36	48	119	84	4448	52.7	33.4
10/18/21	291.6	246.8	28	40	125	112	4305	51.4	32.3
10/25/21	300.1	262.3	34	45	175	128	4539	54.9	35.2
11/01/21	306.9	264.4	37	49	186	125	4264	55.8	33.7
11/08/21	213.4	206.3	37	49	170	113	4125	53.6	31.0
11/15/21	167.9	163.2	48	59	152	116	4054	53.2	32.3
11/20/21	139.2	135.9	54	62	141	139	4442	54.6	32.5

Alb = albumin, normal range 40.0–55.0 g/L, ALP = alkaline phosphatase, normal range 35–100 IU/L, ALT = alanine aminotransferase, normal range 7-40 IU/L, AST = aspartate aminotransferase, normal range 13–35 IU/L, CHE = cholinesterase, normal range 5000–12,000 IU/L, DBil = direct bilirubin, normal range 0.0–8.6 μmol/L, TBil = total bilirubin, normal range 3.4–20.5 μmol/L, TP = total protein, normal range 65.0–85.0 g/L, γ-GT = gamma-glutamyl transpeptidase, normal range 7–45 IU/L.

The patient was concurrently diagnosed with GD and MMI-induced liver injury. In consideration of the fact that the serum levels of FT3 and FT4 were still lower than normal although levothyroxine had been given, she was administered levothyroxine (25 μg/day) consecutively and her thyroid function remains stable during hospitalization (Table 1). She was administered UDCA (500 mg/day), acetylcysteine (8 g/day), and ademetionine (1 g/day). Since no improvement was obtained in liver function, oral treatment with methylprednisolone was added (24 mg/day) on the 16th day after admission. Insulin was administered to control the blood glucose with the initial doses as insulin aspart 4 iu, 4 iu, 4 iu before each meal and were adjusted on the basis of self-monitoring blood glucose (SMBG) levels. After 22 days of methylprednisolone therapy, the bilirubin values fell significantly (TBil 167.9 μmol/L, DBil 163.2 μmol/L) (Table 2). The patient was discharged on the 45th day after admission and continued to methylprednisolone (20 mg/day), UDCA (500 mg/day), levothyroxine (25 μg/day) and aspart (3 iu per meal). Methylprednisolone therapy was gradually tapered by 4 mg/week and discontinued 5 weeks later. Insulin doses were reduced and discontinued accordingly. Her liver function eventually returned to normal one month after discharge and there was no recurrence during a 6-month follow-up. Her thyroid function also returned to normal and levothyroxine was discontinued one month after discharge. The patient’s thyroid function was within normal range during the follow-up of next 3 months, however, laboratory tests revealed low TSH to 0.001 μIU/mL (normal, 0.350–4.940 μIU/mL), elevated FT3 to 5.33 pg/mL (normal, 1.71–3.71 pg/mL), elevated FT4 to 1.58 ng/dL (normal, 0.70–1.48 ng/dL), elevated TRAb to 1.94 U/mL(normal, <1.5 IU/mL) (Table 3) and Radioactive ^99^mTc uptake and scan revealed 2 hours ^99^mTc uptakes was 8.7% (normal, 1.0–2.5%) (Fig. 1) in local hospital about 5 months later which meant her hyperthyroidism reoccurred. Radioactive iodine ablation of the thyroid gland was planned for further treatment of hyperthyroidism.

**Table 3:** Thyroid function tests during follow-up.

Data unit	TT3 ng/mL	TT4 ug/dL	FT3 pg/mL	FT4 ng/dL	TSH μIU/mL
12/21/21	0.99	7.10	2.80	0.83	0.830
01/11/22	1.16	7.70	2.69	0.91	1.016
01/27/22	1.26	7.74	2.90	0.95	1.154
03/22/22	1.37	9.86	3.59	1.15	0.66
04/19/22	2.09	11.89	5.33	1.58	0.001

FT3 = free triiodothyronine, normal range 1.71–3.71 pg/mL, FT4 = free thyroxine, normal range 0.70–1.48 ng/dL, TSH = thyroid stimulating hormone, normal range 0.35–4.94 μIU/mL, TT3 = triiodothyronine, normal range 0.58–1.59 ng/mL, TT4 = tetraiodothyronine, normal range 4.87–11.72 ug/dL.

## 3. Discussion and conclusions

The patient did have hyperthyroidism secondary to GD, with suppression of serum TSH, elevation of serum FT3, FT4, and TRAb. Radioactive ^99^mTc uptake and scan met this diagnosis too. The management of hyperthyroidism became challenging when MMI failed. Total thyroidectomy was not a good treatment choice in our patient for it was contraindicated due to serious liver injury. Therefore, radioactive iodine ablation became the best choice for hyperthyroidism according to Thyroid Association (ETA) guidelines for Diagnosis and Management of Hyperthyroidism and Other Causes of Thyrotoxicosis.^[6]^ However, it was difficult to carry out ^131^I treatment right now for it would damage thyroid acinar cells, increase the release of the thyroxine immediately and aggravate injury on liver function. Therapeutic plasma exchange is a type of blood purification methods to rapidly remove plasma containing pathogenic factors, antibodies and immune complexes, and then resume with fresh plasma. It has been a well-established and effective therapeutic method in the treatment of severe liver injury.^[7,8]^ In addition, it has been reported to successfully used in hyperthyroid patients with antithyroid drug-induced hepatotoxicity.^[3,5]^ Several mechanisms may as followings^[5,9]^: (1) TPE can clear protein-bound thyroid hormones while nearly 99% of the circulating thyroid hormones are bound to serum proteins; (2) TPE can bring new binding sites for free thyroid hormone from replacement with Alb solution to further reduce FT3 and FT4 levels; (3) TPE is also effective to reduce TRAB levels. A decrease in TRAb should play a role in our patient. Unfortunately, information about the variation of TRAb levels before and after each TPE was not available.

Our patient not only suffered from hyperthyroid but also severe liver injury. Liver injury related to hyperthyroidism which can be caused by hyperthyroidism per se, anti-thyroid drugs, and concomitant liver disease.^[10]^ Methimazole is a widely used anti-thyroid drug. However, rare but potentially life-threatening cholestatic jaundice may arise from the use of methimazole. In our patient, the cholestatic jaundice was most likely caused by methimazole. Firstly, there was a tight temporal relationship between initiation of methimazole and onset of cholestatic jaundice 3 weeks later. Secondly, other acquired or inherited causes of liver injury, such as viral hepatitis, autoimmune diseases, Wilson disease, alcohol consumption, and other drugs induced hepatotoxicity were excluded. Thus, she had a RUCAM score of 6^[11]^ and met the diagnosis of MMI-induced liver injury. The pathogenesis of liver injury secondary to MMI remains unclear. Although adverse reactions of MMI are more dose-dependent rather than idiosyncratic, a small number studies showed patients who developed MMI-induced hepatotoxicity had good response to glucocorticoid therapy, indicating an allergic reaction to MMI.^[12]^ In our patient, her cholestatic jaundice was rapidly recovered after about 3-month glucocorticoid therapy without recurrence during a 6-month follow-up, supporting the above findings. Of course, we could not totally ignore the effects of other comprehensive treatment, such as liver-protecting therapy and blood glucose control.

The patients who have severe drug-induced hepatotoxicity related to hyperthyroidism secondary to GD underwent radioactive iodine ablation/surgery or continued oral anti hyperthyroidism medicine immediately after TPE session in the reported literatures.^[3,5]^ However, there has not been any constructive suggestion on the interval between definitive treatments of hyperthyroidism and TPE. An unusual feature of our patient was that her thyroid function was nearly stable after 2 TPE sessions even with levothyroxine (25 μg/day) and euthyroid status persisted after thyroxine discontinuation for a long time, totally about 7 months, without any anti hyperthyroidism treatment. This rare clinical feature has never been reported in literature. We speculate that it may partly relate to the interplay between liver function and thyroid hormones.^[13]^

The liver is not only a target organ of thyroid hormones, but also an important site that produces, excretes, inactivates thyroid hormones, and synthesizes thyroxine-binding globulin. Severe liver damage in patients may result in disordered thyroid hormone deiodination, lower synthesis of thyroxine-binding globulin, and secondary hypothalamic–pituitary–thyroid axis dysfunction,^[14]^ leading to non-thyroid illness syndrome (NTIS) which was characterized by the decreased concentrations of biologic active hormone T3, increased concentrations of inactive hormone rT3, low or normal T4, and normal or mildly decreased concentration of TSH.^[15]^ Based on the above, it is reasonable to infer that our patient is combined with NITS. NTIS is highly prevalent in critical diseases, about 44% to 70% of critically ill patients have suffered from NTIS.^[16]^ The concept that NTIS represents an independent predictor of poorer outcome in critically ill patients is increasingly being consensus.^[17]^ Coronavirus disease 2019 (COVID-19), a new global pandemic with sudden outbreak, rapid spread, and alarming mortality, has drawn lots of attention worldwide. It was reported that approximately 14.47% (34/235) of COVID-19 patients were associated with NTIS to some degree and NITI was also associated with the severity of prognosis of COVID-19.^[18]^ However, should we treat NTIS or not? So far, it is still controversial that thyroid hormone supplement has a positive or negative effect to the clinical outcome of critically ill patients with NTIS.^[19]^ The question remains to be determined in randomized clinical trials with more appropriate primary outcome measures, more appropriate types, dosages, duration and administration pathways of thyroid hormone supplementation.^[19]^ Since our patient had been treated with levothyroxine (25 μg/day) before admission and the levels of FT3 and FT4 were still lower than normal, we continued the above treatment. We conducted a rigorous follow-up and found that her thyroid function gradually returned to normal as her liver function and overall condition improved.

On the other hand, our patient was given methylprednisolone. It is known that the administration of exogenous glucocorticoids has inhibitory effect on the pituitary and it is difficult to distinguish between severe NTIS and central hypothyroidism,^[20]^ so the observed abnormalities such as decreased TSH, FT3, and FT4 in our patient may be also partially explained by the use of exogenous glucocorticoids.

In conclusion, we report a case of hyperthyroidism secondary to GD overlapping with severe MMI-induced liver injury and type 2 diabetes. We propose that NTIS and partial central hypothyroidism status made the thyroid function stable for a long time after TPE without any anti hyperthyroidism treatment. From our case, careful and individualized follow-up is especially important as the patient began to approach the appropriate therapy.

## Author contributions

**Conceptualization:** Xiaohong Feng.

**Data curation:** Yamei Jin.

**Funding acquisition:** Yamei Jin.

**Supervision:** Haixiang Ni.

**Writing – original draft:** Yamei Jin.

**Writing – review & editing:** Yamei Jin, Xiaohong Feng, Haixiang Ni.
